# Long-Range Correlations and Memory in the Dynamics of Internet Interdomain Routing

**DOI:** 10.1371/journal.pone.0141481

**Published:** 2015-11-03

**Authors:** Maksim Kitsak, Ahmed Elmokashfi, Shlomo Havlin, Dmitri Krioukov

**Affiliations:** 1 Department of Physics, Northeastern University, Boston, MA, United States of America; 2 Simula Research Lab, Oslo, Norway; 3 Department of Physics, Bar-Ilan University, Ramat Gan, Israel; 4 Department of Mathematics, Northeastern University, Boston, MA, United States of America; 5 Department of Electrical&Computer Engineering, Northeastern University, Boston, MA, United States of America; University of Warwick, UNITED KINGDOM

## Abstract

Data transfer is one of the main functions of the Internet. The Internet consists of a large number of interconnected subnetworks or domains, known as Autonomous Systems (ASes). Due to privacy and other reasons the information about what route to use to reach devices within other ASes is not readily available to any given AS. The Border Gateway Protocol (BGP) is responsible for discovering and distributing this reachability information to all ASes. Since the topology of the Internet is highly dynamic, all ASes constantly exchange and update this reachability information in small chunks, known as routing control packets or BGP updates. In the view of the quick growth of the Internet there are significant concerns with the scalability of the BGP updates and the efficiency of the BGP routing in general. Motivated by these issues we conduct a systematic time series analysis of BGP update rates. We find that BGP update time series are extremely volatile, exhibit long-term correlations and memory effects, similar to seismic time series, or temperature and stock market price fluctuations. The presented statistical characterization of BGP update dynamics could serve as a basis for validation of existing and developing better models of Internet interdomain routing.

## Introduction

On large scale, the Internet is a global system of approximately 40,000 interlinked computer networks connecting billions of users and devices worldwide [1]. These networks are called Autonomous Systems (ASes). ASes vary in size and function: they can be (i) Internet Service and/or Transit Providers (*AT*&*T*), (ii) Content Providers (Google), (iii) Enterprises (Harvard University), and (iv) Non-profit organizations [2]. Devices inside ASes are identified via unique Internet Protocol (IP) addresses, which are 32- or 128-bit numerical labels that act both as identifiers and locators of devices. An IP address is divided into two sections, a network section and a host section. The network section, which is known as IP prefix, identifies a group of hosts, while the host section identifies a particular device. An AS can include a number of IP prefixes.

**Fig 1:**
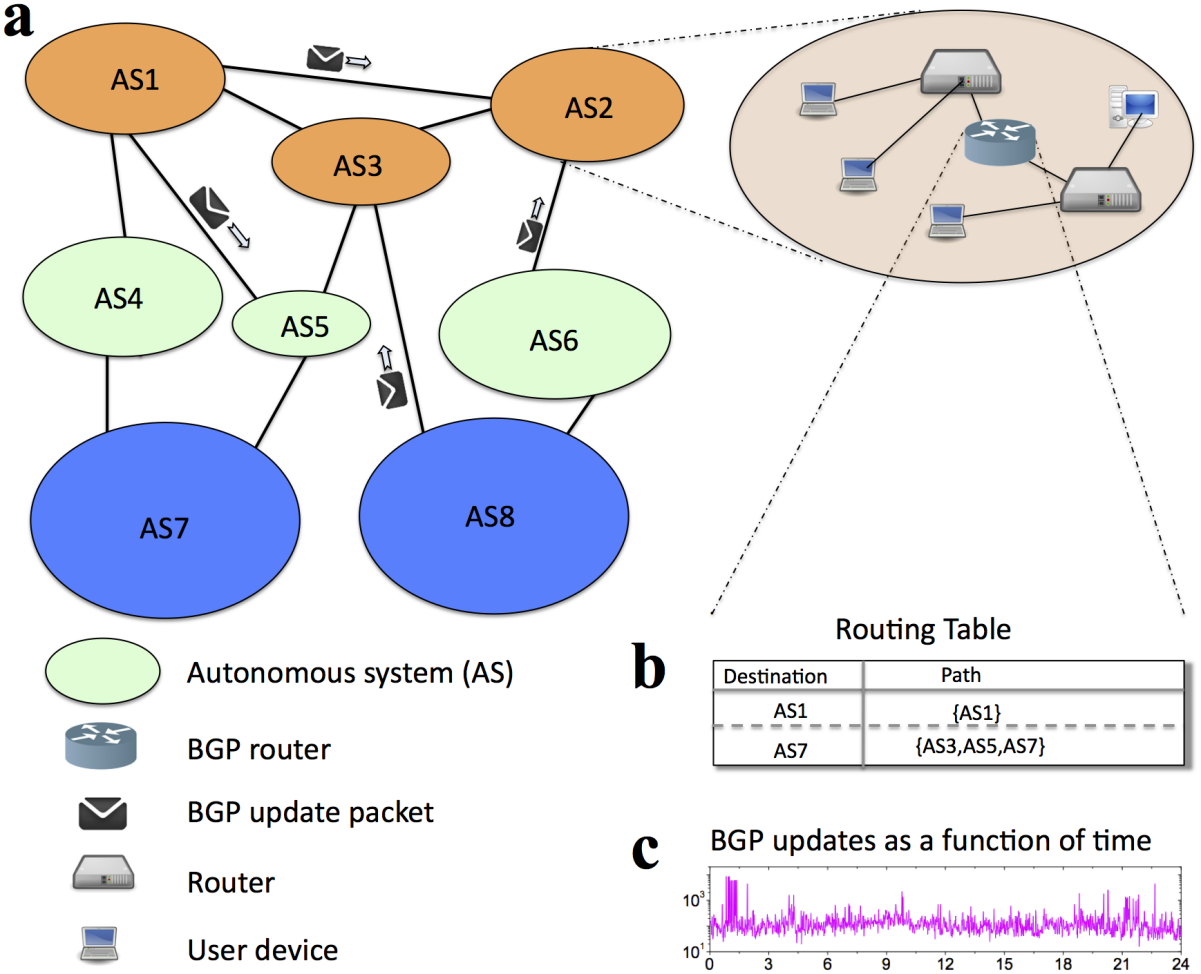
The Internet and BGP routing. **a**, On large scale the Internet is the product of interconnectivity among a large number of ASes (shown with ovals). In order to perform data transfer, ASes need to exchange the reachability information through BGP update messages. **b, c** BGP updates are processed by the BGP routers. **b**, The reachability information is stored in the routing table. **c**, Typical dynamics in the number of updates received by the BGP router.

Each AS is administrated by a single entity, but a single organization may own and operate several ASes. ASes connect to each other via contractual agreements that govern the flow of data between and through them. This interconnection of ASes shapes the AS-level topology of the Internet, which facilitates connectivity between any pair of ASes and thus any pair of devices connected to the Internet (Fig 1**a**).

The information about how to reach devices within other ASes is not readily available to them. The exchange of this information is handled by specialized networked computers called routers. Performing routing requires signaling reachability information, comparing different possibilities, and maintaining a state that describes how to reach different IP prefixes. The Border Gateway Protocol (BGP) [3] is the globally deployed routing protocol that accomplishes this task. The BGP protocol can be summarized as follows. ASes advertise their IP prefixes to their neighbor ASes through BGP update messages. At each AS incoming BGP updates are processed by the BGP router and the resulting reachability information is then stored in routing tables.

The Internet is a dynamic system where participating networks and links between them do often experience configuration changes, failures, and restorations. BGP protocol reacts to changes in the Internet connectivity incrementally: BGP routers send update messages to their neighbor BGP routers. BGP update messages do not carry the information on the whole Internet connectivity state. Instead, they carry only the information concerning the affected IP prefixes. Hence, to keep a consistent view of the network and, consequently, to be able to communicate with other networks, a BGP router must process incoming BGP updates in a timely manner and update its routing table accordingly (Fig 1**b** and 1**c**).

Current version of the BGP routing protocol was introduced in 1994. Since then, the deployment of the BGP routing protocol has sustained tremendous growth and it is arguably one of the main technological reasons behind the success of the Internet.

Nevertheless, there are two major concerns related to the fast rate of the Internet growth. On one hand, Internet growth implies the growth in the number of destinations for the BGP routing and, thus, results in the growth of routing table sizes. On the other hand, the growth of the Internet also leads to the growth in the number of BGP updates needed to maintain BGP routing [4]. Both factors are important, especially for routers at the core of the Internet. The growing size of routing tables requires increasingly larger and faster memory. At the same time, growing routing table sizes do not necessarily slow down data forwarding as long as address lookups are performed using high speed memories and constant-time matching algorithms [5]. Increasingly large amounts of BGP updates, on the other hand, is a more serious concern because processing BGP updates can be computationally heavy (updating routing state, generating more updates, checking import/export filters), and can trigger wide-scale instabilities [6].

Recent studies of BGP scalability range from measurements assessing the extent of the concern [7, 8] to studies suggesting radically new routing architectures [9, 10]. Elmokashfi et al. [7] analyzed the dynamics of BGP updates in four networks at the backbone of the Internet over a period of seven years and eight months. They have shown that on average the level of BGP updates is increasing, but not at an alarming rate: it was shown to grow at rates similar to the growth in the number of ASes. However, they have also illustrated that the dynamics of BGP updates is highly volatile even at large time scales, with peak rates exceeding the daily averages by several orders of magnitude.

The complexity of the inter-AS routing system makes it difficult to isolate different factors behind these fluctuations [11, 12]. An approach alternative to inferring this factors directly is to build a realistic model for the dynamics of BGP updates. To this end, one needs an in-depth statistical characterization of fluctuations in BGP update time series, which is the subject of this work.

We aim at improving our understanding of these fluctuations, which can help in validating existing models [13] and in developing better ones. To study the statistical properties of BGP updates, we use historical BGP update logs spanning a period of 8.5 years, collected by the RouteViews project [14] from the BGP routers of four ASes (*AT*&*T*, *NTT*, *IIJ*, and *Tinet*). Throughout the manuscript we refer to these routers as monitors. A BGP update log is the time series of BGP updates arriving at the monitor recorded in 1 second intervals. The four ASes analyzed in this work are among the largest Internet Service Providers (ISPs). Therefore, their corresponding BGP update traffic is a reflection of BGP dynamics taking place in the core of the Internet, where the BGP update volatility is believed to reach maximum rates. (Detailed information on data collection and pre-processing can be found in the S1 Text Section II).

To put our study in a broader context we wish to note that many natural and economic systems have also been found to exhibit extreme fluctuations. Examples include DNA sequences [15] and heartbeat intervals [16], climate variability [17, 18], earthquakes [19, 20], stock markets [21–23], and languages [24, 25].

## Analysis

**Fig 2:**
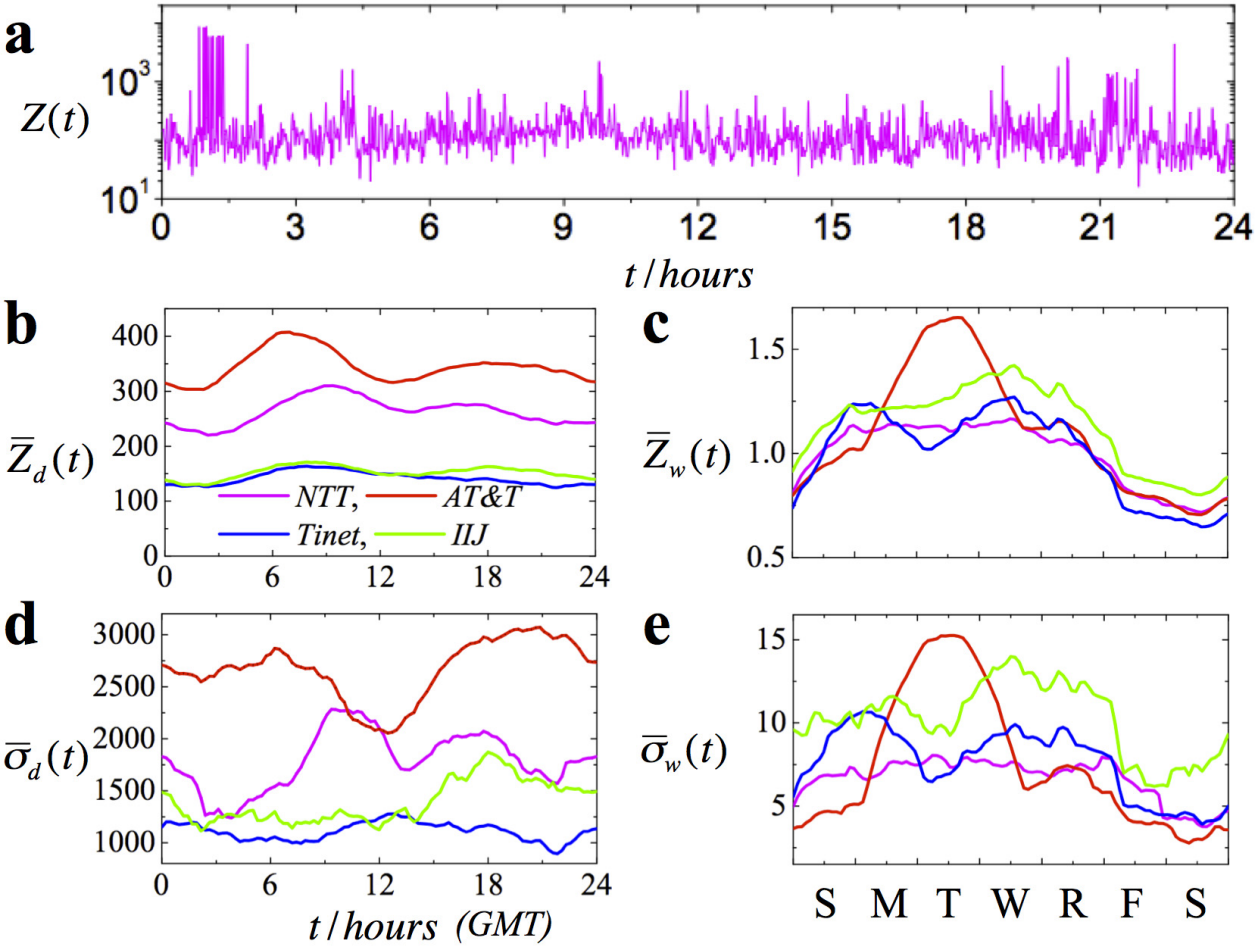
Time series of BGP updates. **a,** The number of updates received by the *NTT* monitor on May 28th, 2010, from 00.00 to 24.00 GMT. **b**, the intra-day pattern, ${\bar{Z}}_{d}\left(t\right)$, and, **d**, the standard deviation from the intra-day pattern, ${\bar{\sigma }}_{d}\left(t\right)$, of BGP updates measured for *NTT*, *IIJ*, *Tinet*, and *AT*&*T* monitors. **c**, the intra-week pattern, ${\bar{Z}}_{w}\left(t\right)$, and, **e**, the standard deviation from the intra-week pattern, ${\bar{\sigma }}_{w}\left(t\right)$, of BGP updates measured for *NTT*, *IIJ*, *Tinet*, and *AT*&*T* monitors.

First we highlight the volatility of BGP updates series by reproducing the results of previous works [7]. We plot the average rate of BGP updates received by the *NTT* monitor on May 28th, 2010, from 00: 00 to 24: 00 Greenwich Mean Time (GMT). As seen from Fig 2**a**, in 1 minute interval the *NTT* monitor receives on the average several hundred updates, while extreme fluctuations occasionally produce 10^4^ updates per minute. BGP updates are largely driven by two sources: spontaneous BGP events and maintenance sessions. The former consist of mostly spontaneous updates, such as misconfigurations, duplicate announcements and special events. Maintenance sessions, on the other hand, are periodic by nature and happen at certain times of the day on particular days of the week.

In order to separate the two sources of fluctuations we calculate the intra-day and intra-week patterns for the BGP update time series. The intra-day pattern, ${\bar{Z}}_{d}$, is then defined as the number of events taking place at a specific time of the day, *t*_*day*_, averaged throughout the observation period:

$${\bar{Z}}_{d}\left(t_{day}\right)=\frac{1}{N_{d}}\sum_{i=1}^{N_{d}}Z^{i}\left(t_{day}\right),$$ (1)

where *N*_*d*_ is the total number of days in the observation period, and *Z* ^*i*^(*t*_*day*_) is the number of events at day *i* at *t*_*day*_. The intra-week pattern ${\bar{Z}}_{w}\left(t_{week}\right)$ is defined in a similar way after first normalizing the time series with the intra-day pattern.

$$\begin{array}{ccc} \tilde{Z}\left(t\right) & \equiv & \frac{Z(t)}{{\bar{Z}}_{d}\left(t_{day}\left(t\right)\right)}, \end{array}$$ (2)

$$\begin{array}{ccc} {\bar{Z}}_{w}\left(t_{week}\right) & = & \frac{1}{N_{w}}\sum_{i=1}^{N_{w}}{\tilde{Z}}^{i}\left(t_{week}\right) \end{array}$$ (3)

Here *N*_*w*_ is the number of weeks in the observational period and ${\tilde{Z}}^{i}\left(t_{week}\right)$ is the normalized number of events at week *i* at time of the week *t*_*week*_ (see Methods for details).

As seen from Fig 2**b**, the intraday BGP update patterns reach maximum values in the interval from approximately 06: 00 to 10: 00 GMT, which is typical time for scheduling maintenance tasks [26]. The intraweek patterns, in their turn, are characterized by higher values during weekdays and smaller values during weekends. (see Fig 2**c**). We note that the standard deviations of the intraday and intraweek patterns, ${\bar{\sigma }}_{d}\left(t\right)$ and ${\bar{\sigma }}_{w}\left(t\right)$, tend to exceed the corresponding average values of the intra-day and the intra-week patterns by an order of magnitude, which is consistent with the extreme burstiness of the BGP updates (Fig 2**d** and 2**e**).

**Fig 3:**
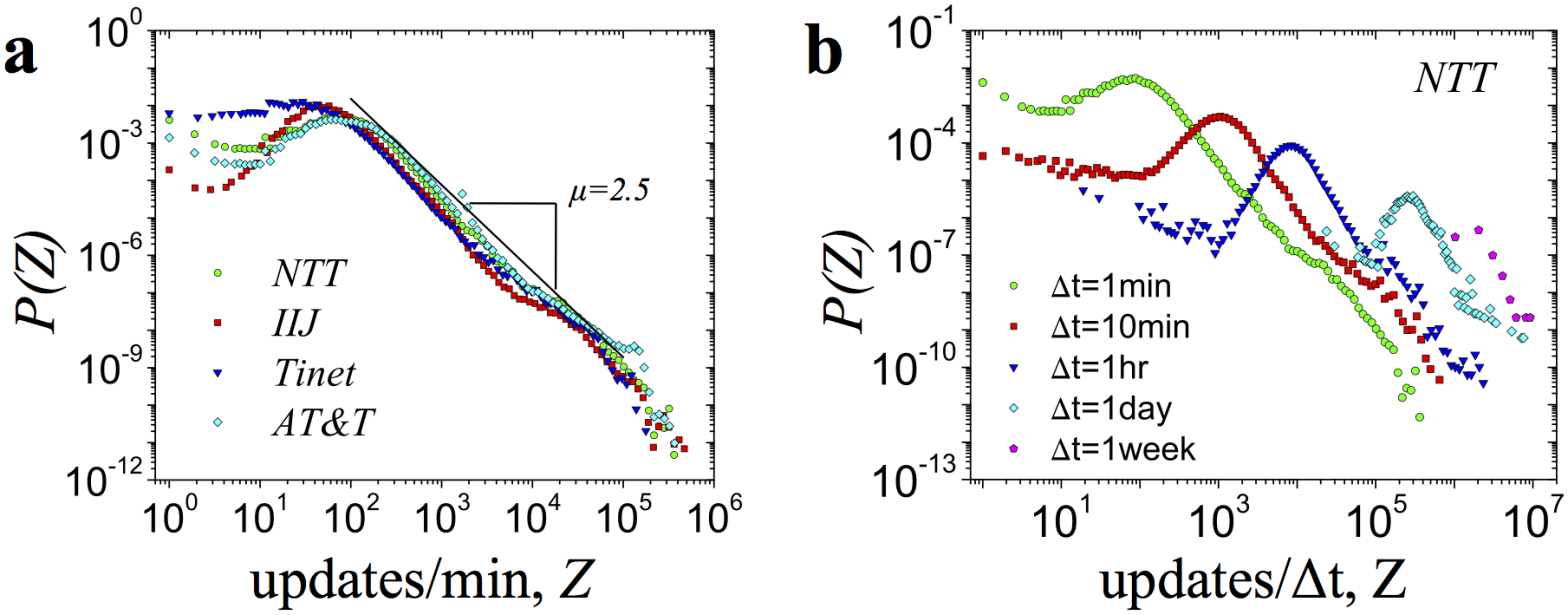
Extreme events in BGP dynamics. **a**, The distribution of the number of BGP updates received by the 4 monitors in 1 minute interval, All monitors collapse onto a single master curve. Power law regression fit yields a slope of *μ* = 2.3 **b**, The distribution of number of updates *P*(*z*) received by the *NTT* monitor calculated for aggregation window sizes Δ*t* = 1*min*, Δ*t* = 10*min*, Δ*t* = 1*hour*, Δ*t* = 1*day* and Δ*t* = 1*week*.

To characterize the volatility of the BGP updates we analyze the distribution of the number of BGP updates received by the monitor in 1 minute intervals. Fig 3**a** confirms the volatile nature of BGP updates. We find that all monitors are characterized by similar distributions *P*(*Z*). Although the average number of BGP updates received per minute is quite small (${\bar{Z}}_{NTT}=250$), the peak values may occasionally exceed 10^5^ BGP updates per minute. The distributions of the number of BGP updates, *P*(*Z*), are positively skewed (measured skewness values are: *γ*_1_(AT&T) = 45.7, *γ*_1_(*IIJ*) = 121.2, *γ*_1_(*Tinet*) = 49.1, *γ*_1_(*NTT*) = 69.1) and the distribution tails scale as a power-law, *P*(*Z*) ∼ *Z* ^−*μ*^ with *μ* = 2.51 ± 0.11 (*p* = 0.992 for *IIJ*, see S1 Text Section V for details). We also note that the observed power-law behavior of the tail of *P*(*Z*) seems to be independent of the aggregation window size (Fig 3**b**).

The power-law distribution of the number of BGP updates implies that BGP routers should be able to cope with surges in the number of updates exceeding the corresponding average levels by several orders of magnitude. To understand how and when these surges occur we analyze correlation patterns of the BGP updates. We employ three standard methods traditionally used in the time-series analysis: auto-correlation function (ACF), power spectrum (PS), and the linear detrended fluctuation analysis (DFA1) (see Methods, S1 Text Section IV, and Ref. [27] for details).

Even though most of BGP update events last less then 1 minute, the duration of some of them may exceed several minutes [28]. Thus, to avoid possible correlations associated with long BGP updates in our subsequent analysis we use larger aggregation window size of Δ(*t*) = 10*min*. Further, to eliminate possible spurious effects and correlations attributed to periodic activities we also normalize the BGP update data with both intra-week and intra-day patterns:

$$z\left(t\right)\equiv \frac{Z(t)}{{\bar{Z}}_{w}\;\left(t_{week}\left(t\right)\right){\bar{Z}}_{d}\;\left(t_{day}\left(t\right)\right)},$$ (4)

**Fig 4:**
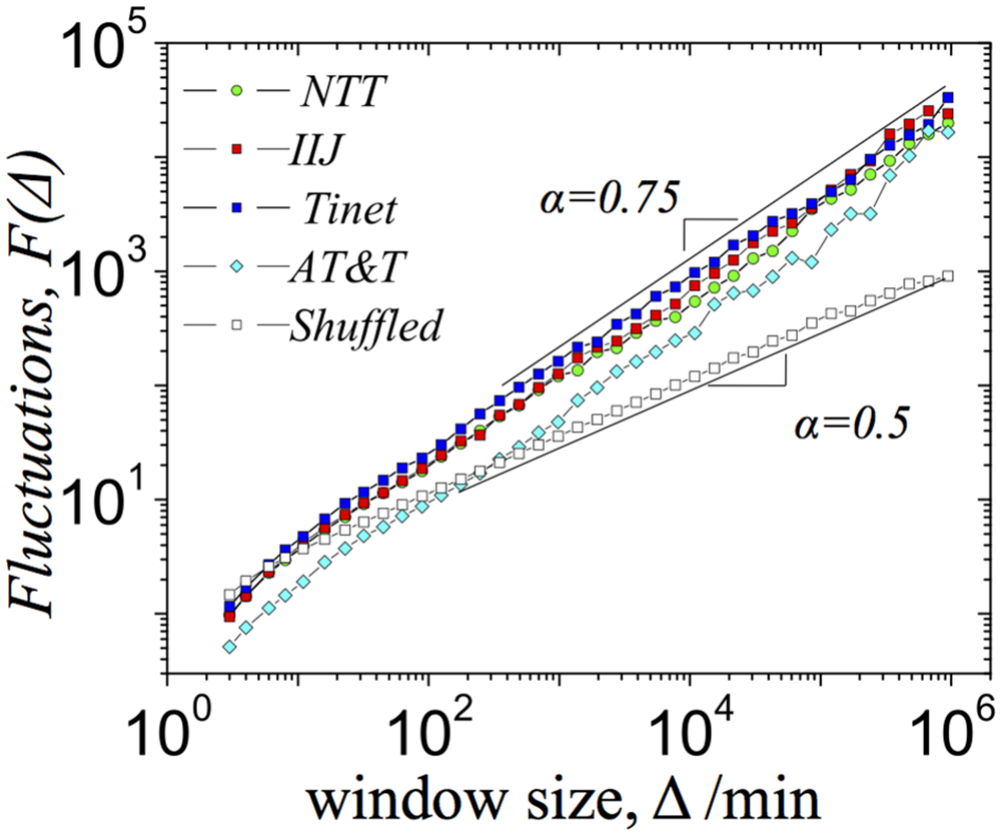
Correlations in the BGP update times series. Fluctuations of the detrended BGP update time series as a function of window size.

All three methods indicate the presence of long-range correlations in the BGP update time-series (see Fig 4 and S1 Fig). Specifically, we find that DFA1 performed for *NTT*, *IIJ* and *Tinet* and *AT*&*T* indicates that fluctuations grow as a power-law with aggregation window size Δ, *F*(Δ) ∼ Δ^*α*^, where *α* = 0.75 (Fig 4). To highlight the effects of long-range correlations in the BGP updates time series we also performed DFA1 for the randomized counterparts of the BGP updates (see Methods). In the randomized case we obtained *F*_*random*_(Δ) ∼ Δ^*α*^ with *α* = 0.5, which corresponds to the uncorrelated time series (Fig 4). Similar results are obtained by ACF and PS analysis. The autocorrelation function of the BGP updates decays as a power law over several orders of magnitude for all monitors, *ACF*(Δ*z*) ∼ *z* ^−*γ*^ (S1**a** Fig). We obtain similar *γ* values for three monitors: *γ* = 0.5 for *NTT*, and *γ* = 0.4 for *IIJ* and *Tinet* monitors. The power spectrum density, *S*(*f*), also decays as a power-law with frequency, *S*(*f*) ∼ *f* ^−*β*^, where *β* = 0.6 for all monitors (S1**b** Fig). We note that the obtained values of correlation exponents approximately conform with expected relations, *γ* = 1 − *β*, $\alpha =\frac{\beta +1}{2}$, and *γ* = 2(1 − *α*) [17, 29–31].

**Fig 5:**
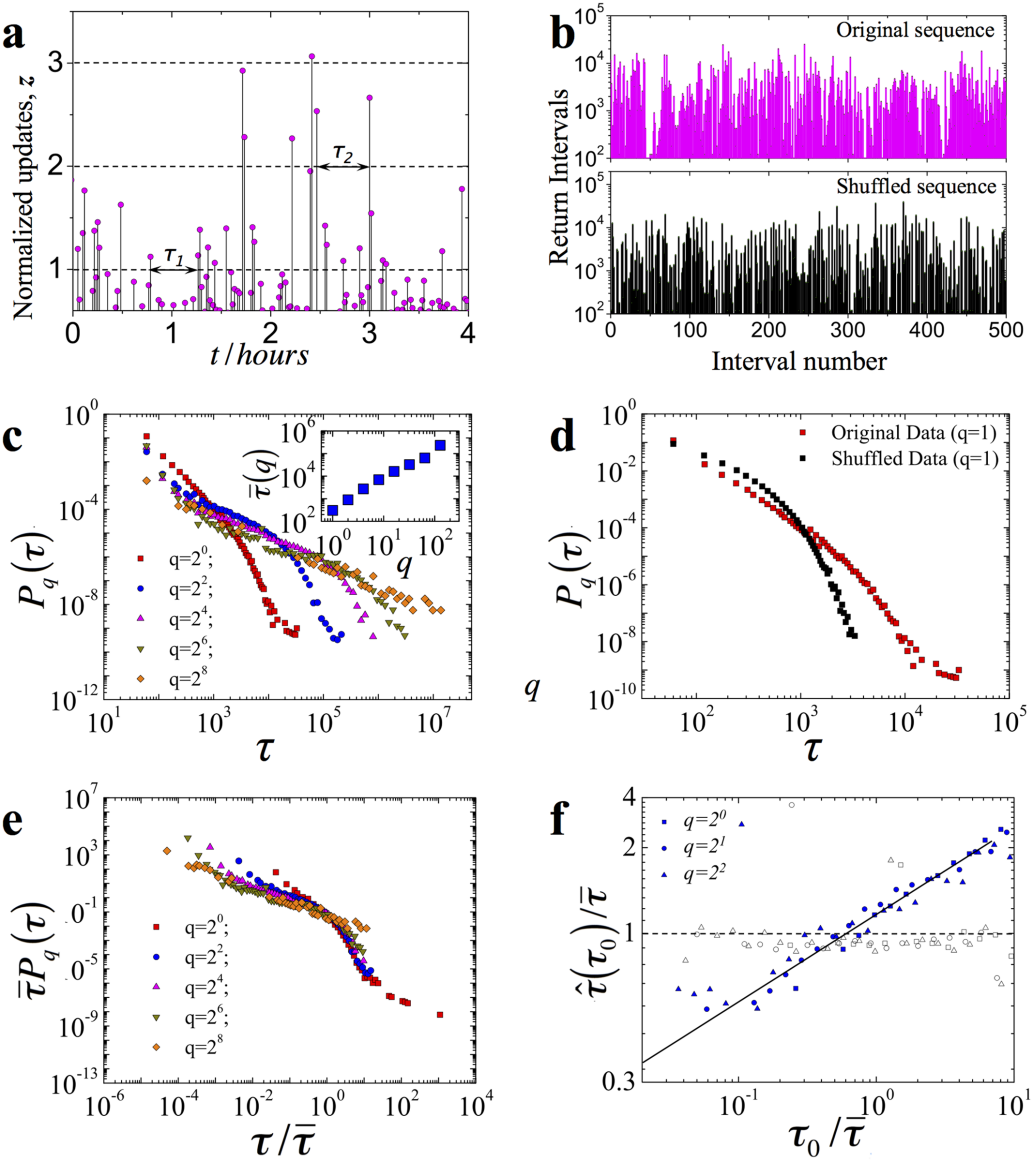
Return interval statistics of BGP updates. **a**, Schematic illustration of the BGP update return intervals. Shown are the intervals *τ*_1_ and *τ*_2_ calculated for threshold *q* = 1 and *q* = 2 respectively. **b**, Typical sequence of 500 BGP update return intervals for *NTT*, where *q* = 4, calculated for (magenta) original and (black) shuffled data. **c**, The distribution function *P*_*q*_(*τ*) of BGP update return intervals of the *NTT*, calculated for different values of *q*. The inset depicts the average return interval $\bar{\tau }$ as a function of threshold *q*. **d**, *P*_*q*_(*τ*) for BGP update return intervals of the *NTT* monitor calculated for *q* = 1. Original data is shown with red while shuffled data is shown with black. **e**, Scaled plots of the BGP return intervals for the *NTT* monitor. **f**, The mean conditional return interval $\hat{\tau }$ as a function of preceding return interval *τ*_0_ for the *NTT* monitor. Both $\hat{\tau }$ and *τ*_0_ are normalized with the mean return interval ($\bar{\tau }$). For BGP updates without memory we expect $\hat{\tau }\left(\tau_{0}\right)=1$, as supported by the open symbols obtained for shuffled return interval data.

The appearance of long-range correlations in BGP update time series indicates that at a given time the state of a particular BGP router is determined by its previous states. Consequently, long-range correlations may imply the presence of memory effects in the inter-domain Internet routing. To probe for the latter we ask, what is a typical time interval *τ* separating two large events. Formally, we define a return interval *τ*(*q*) as a time separation between two consecutive events *z*(*t*_1_) and *z*(*t*_2_), such that *z*(*t*_1_) > *q* and *z*(*t*_2_) > *q* (see Fig 5**a**). The evidence of memory in BGP update time series is seen in Fig 5**b**, which displays typical sequence of 500 consecutive return intervals for the *NTT* monitor. The original return interval data (shown in magenta) is characterized by “patches” of extreme return intervals, while there is no such “patches” in the shuffled data (shown in black) obtained by randomizing the time-order of the original series of BGP updates.

To further explore memory effects we analyze the distribution of return intervals *P*_*q*_(*τ*) for the *NTT* monitor (Fig 5**c**). We note that *P*_*q*_(*τ*) decays slower than the Poisson distribution, which is expected for uncorrelated data (Fig 5**d**). As *q* increases, the decay of *P*_*q*_(*τ*) becomes slower and the average return interval $\bar{\tau }\left(q\right)$ increases implying that the larger events become increasingly rare (see the inset of Fig 5**c**)). We also note that, independent of *q*, all the distributions *P*_*q*_(*τ*), upon proper rescaling, collapse to a single master curve:

$$P_{q}\left(\tau \right)=\frac{1}{\bar{\tau }}f\;\left(\frac{\tau }{\bar{\tau }}\right),$$ (5)

where *f*(*x*) does not depend on the threshold value (see Fig 5**e** and S3 Fig). The resulting master curve *f*(*x*) fits a stretched exponential *exp*(−*x* ^−*γ*^) with exponent *γ* = 0.5 (*p* = 0.08, see S1 Text Section V), which approximately matches the observed autocorrelation exponent *γ* = 0.5 [32]. We note that the observed scaling of *P*_*q*_(*τ*) holds not only for *NTT* but also for the other three analyzed monitors (see S1 Text Section IV and S2 Fig).

Finally, to test memory effects directly we measure the average return interval $\hat{\tau }$ following immediately after return intervals of fixed duration *τ*_0_. Fig 5**f** and S3 Fig depict $\hat{\tau }$ as a function of *τ*_0_ for three possible values of threshold *q* (filled symbols). We observe that $\hat{\tau }$ increases as a function of *τ*_0_, indicating that on the average longer (shorter) return intervals tend to follow longer (shorter) intervals. In contrast, $\hat{\tau }$ is independent of preceding return interval *τ*_0_ for randomized data (open symbols in Fig 5**f** and S3 Fig).

## Discussion

In this work, we investigated the statistical properties of BGP updates. Complementing previous studies, we confirmed that the rate of BGP updates is highly volatile, with extreme events at times exceeding the average rates by up to 4 orders of magnitude. We established that the distribution in the number of BGP updates received by a BGP monitor in a given time window is characterized by a power-law tail with exponent *μ* = 2.5. We also found (using three independent methods) that the BGP update time series exhibit long-range correlations. The analysis of the return interval data revealed the universal scaling in the distribution of return intervals *P*_*q*_(*τ*). We also found memory effects in the return interval data. Small (or large) return intervals separating BGP update events are more likely to be followed by small (or large) intervals.

The observed volatility and correlation properties of the BGP update dynamics place interdomain Internet routing into the same class of phenomena as earthquakes [19, 20], climate [18], stock markets [21–23] and languages [24, 25]. Unlike these systems, however, the Internet routing is a fully engineered system. The observed dynamical similarities between these stochastic systems imply that the key mechanisms underlying Internet routing are in a certain way similar to the mechanisms governing the dynamics of stock markets or seismic movements in the Earth crust.

As with stock market price dynamics, one would wish to be able to predict BGP dynamics, or at least extreme events in it. To this end, one could benefit from the return interval scaling. The established scaling of *P*_*q*_(*τ*) may allow one to approximate the statistics of return intervals for large events (characterized by large *q* values) using the much richer statistics of return intervals of smaller events.

The observed long-range correlations and memory effects indicate that the communication patterns between BGP routers are an outcome of an interplay between certain semi-deterministic processes. Such processes are well known at the low level of the operation of an individual BGP router (e.g. BGP route selection process). Yet this knowledge is as helpful as the knowledge about the dynamical properties of an individual molecule in a gas—when studying the properties of this gas (or the Internet in our case), some molecular details do matter, but most details are irrelevant.

Therefore the identification of a proper level of abstraction in modeling the dynamics of BGP routing is an important problem for understanding Internet dynamics. The statistical analysis of the BGP update time series that we have conducted here should serve as a basis for validation of existing models and for developing better ones.

## Materials and Methods

### Intraday and Intraweek Patterns

Consider series *Z*(*t*), where *Z* is the number of events taking place at time *t*, and *t* is specified as UNIX timestamps. We first define functions *t*_*day*_(*t*) and *t*_*week*_(*t*) which map Unix timestamps *t* to respectively specific time of the day or specific time of the week (*t_day_* ∈ [0: 00, 24: 00], *t_week_* ∈ [*Sunday*, 0: 00, *Saturday*, 24: 00]). Both *t*_*day*_ and *t*_*week*_ are calculated corresponding to the GMT time zone.

The intra-day pattern, ${\bar{Z}}_{d}$, is then defined as the number of events taking place at a specific time of the day, *t*_*day*_, averaged throughout the observation period:

$${\bar{Z}}_{d}\left(t_{day}\right)=\frac{1}{N_{d}}\sum_{i=1}^{N_{d}}Z^{i}\left(t_{day}\right),$$ (6)

where *N*_*d*_ is the total number of days in the observation period, and *Z* ^*i*^(*t*_*day*_) is the number of events at day *i* at *t*_*day*_. The intra-week pattern ${\bar{Z}}_{w}\left(t_{week}\right)$ is defined in a similar way after first normalizing the time series with the intra-day pattern.

$$\begin{array}{ccc} \tilde{Z}\left(t\right) & \equiv & \frac{Z(t)}{{\bar{Z}}_{d}\left(t_{day}\;\left(t\right)\right)}, \end{array}$$ (7)

$$\begin{array}{ccc} {\bar{Z}}_{w}\left(t_{week}\right) & = & \frac{1}{N_{w}}\sum_{i=1}^{N_{w}}{\tilde{Z}}^{i}\left(t_{week}\right). \end{array}$$ (8)

Here *N*_*w*_ is the number of weeks in the observational period and ${\tilde{Z}}^{i}\left(t_{week}\right)$ is the normalized number of events at week *i* at time of the week *t*_*week*_.

The standard deviations of the intraday and intraweek patterns are defined as

$$\begin{array}{ccc} \sigma_{d}\left(t_{day}\right) & \equiv & \sqrt{\frac{1}{N_{d}}\sum_{i=1}^{N_{d}}\left(Z^{i}\left(t_{day}\right)-{\bar{Z}}_{d}{\left(t_{day})\right)}^{2}\right.}, \end{array}$$ (9)

$$\begin{array}{c} \sigma_{d}\left(t_{week}\right)\equiv \sqrt{\frac{1}{N_{w}}\sum_{i=1}^{N_{w}}\left({\tilde{Z}}^{i}\left(t_{week}\right)-{\bar{Z}}_{w}{\left(t_{week})\right)}^{2}\right.} \end{array}$$ (10)

### Detrended Fluctuation Analysis

Detrended Fluctuation Analysis is a method designed to study correlations in time series [27]. Here we employ the linear version of the DFA, defined as follows. We first calculate the cumulative BGP update time series:

$$y\left(t\right)=\sum_{t^{\prime }=t_{i}}^{t}\left(z\left(t^{\prime }\right)-\bar{z}\right),$$ (11)

where *t*_*i*_ is the initial time value in the series, *z*(*t*) is the original time series and $\bar{z}$ is its average value. The cumulative time series *y*(*t*) is then divided into boxes of equal size Δ. In each box, a least squares linear fit to the *y*(*t*) data is performed, representing the trend in that box. That is, for each box Δ we determine linear approximation for the corresponding piece of the time series:

$$y_{\Delta }\left(t\right)=m_{\Delta }t+b_{\Delta },$$ (12)

where *m*_Δ_ and *b*_Δ_ are the slope and the intercept of the straight line. Next we detrend the integrated time series, *y*(*t*), by subtracting the local trend, *y*_Δ_(*t*), in each box. The root-mean-square fluctuation of this integrated and detrended time series is calculated:

$$F\left(\Delta \right)=\sqrt{\frac{1}{N}\;\sum_{t=t_{i}}^{t_{f}}{\left[y\left(t\right)-y_{\Delta }\left(t\right)\right]}^{2}},$$ (13)

where *N* is the total number of points in the original time series, *t*_*i*_ and *t*_*f*_ are respectively the initial and final time values in the series.

This fluctuation measurement process is repeated at a range of different box sizes Δ. The fluctuations typically exhibit a power law scaling as a function of box size:

$$F\left(\Delta \right)∼\Delta^{\alpha },$$ (14)

depending on the observed exponent *α* one can distinguish anti-correlated fluctuations (*α* < 1/2), uncorrelated fluctuations (*α* = 1/2), and correlated fluctuations (*α* > 1/2).

### Data Randomization

To assess the significance of correlations and memory effects in the BGP update time series we compare original results to those obtained for randomized (shuffled) datasets. In all experiments the randomization is performed at the most basic level: for a given time series *Z*(*t*) we obtain its randomized (shuffled) counterpart by randomly rearranging time stamps attributed to each element in the series. Shuffled data is subsequently normalized and binned using the same procedures as those applied to original data.

## Supporting Information

S1 Text (PDF)

S1 Fig Correlations in the BGP update times series. **a**, The autocorrelation function, ACF and **b**, The Power Spectrum *S*(*f*).
(TIFF)

S2 Fig (Left column) The distribution of return intervals, *P*_*q*_(*τ*), for BGP updates of **a**, *NTT*, **c**, *AT*&*T*, **e**, *Tinet*, and **g**, *IIJ* monitors. The distributions are calculated for different values of threshold *q*. (Right column) Rescaled plots of the BGP return intervals of **b**, *NTT*, **d**, *AT*&*T*, **f**, *Tinet*, and **h**, *IIJ* monitors.
(TIFF)

S3 FigThe mean conditional interval $\hat{\tau }\left(\tau_{0}\right)$ divided by $\bar{\tau }$ as a function of $\frac{\tau_{0}}{\bar{\tau }}$ for **a** *NTT*, **b** *AT*&*T*, **c** *Tinet*, and **d** *IIJ* monitors. In time series without memory, $\hat{\tau }\left(\tau_{0}\right)=1$, indicated by the open symbols that show the shuffled return interval data.(TIFF)

S4 Fig KS goodness of fit tests for **a**, the distribution of number of BGP updates, *P*(*z*) for the *NTT* monitor, and **b**, the distribution of return intervals *P*_*q*_(*τ*) for the *NTT* monitor.
(TIFF)
