# Supplementary material for: Long-Range Correlations and Memory in the Dynamics of Internet Interdomain Routing
Source: PLoS One. 2015 Nov 3;10(11):e0141481. doi: 10.1371/journal.pone.0141481 (PMC4631327; doi:10.1371/journal.pone.0141481)
Supplement: S1 Text — (PDF) [file pone.0141481.s001.pdf]

# Supporting Information:
# Long-Range Correlations and Memory in the Dynamics of Internet Interdomain Routing

Maksim Kitsak, Ahmed Elmokashfi, Shlomo Havlin, and Dmitri Krioukov

## I. INTRODUCTION

This document is organized as follows: In Section II we describe BGP monitors and the process of BGP update data collection and pre-processing. In section III we discuss methods used to measure BGP update series correlation patterns. In section IV we provide the results of the return interval analysis performed for the *AT&T*, *Tinet*, and *IIJ* monitors. Finally, in section V we report the results of the Kolmogorov Smirnov goodness of fit tests.

## II. THE COLLECTION AND PRE-PROCESSING OF THE BGP UPDATE DATA

Our analysis is based on BGP update traces collected by the RouteViews project [1]. Routeviews collects the BGP update data from select ASes. BGP routers in these ASes are referred to as *monitors*. Monitors send BGP updates to the Routeviews collector every time there is a routing change. BGP updates are recorded by the Routeviews with a granularity of one second. We focus on update traces from monitors at large transit networks in the core of the Internet. Specifically, we analyze the BGP update time series from four monitors: *AT&T*, *NTT*, *IIJ*, and *Tinet*.

*AT&T* (American Telephone & Telegraph) is an American multinational telecommunications corporation, headquartered in Dallas, TX. *AT&T* is one of the largest providers of telephone services in the United States. *AT&T* also provides broadband subscription to television services. *NTT* (Nippon Telegraph and Telephone) is a Japanese telecommunications company headquartered in Tokyo, Japan and is one of the largest telecommunications companies in the world in terms of revenue. *IIJ* (Internet Initiative Japan) is the first Japan's Internet provider, which is headquartered in Tokyo, Japan. *IIJ* is currently known as total solutions provider, offering network services, value-added outsourcing services, and cloud computing, WAN services and systems integration services. *Tinet* (The Tiscali International Network) is an Italian Internet service provider headquartered in Cagliari, Italy. *AT&T*, *NTT*, and *Tinet* are present worldwide. *IIJ* has very strong presence in Japan and also operates in the US, UK, Germany, China, Hong Kong, Indonesia, Singapore, and Thailand.

*AT&T*, *NTT*, *IIJ*, and *Tinet* monitors correspond to the largest Internet Service Providers and belong to the *Default Free Zone*. In other words, they have a route to every destination prefix on the Internet. Thus, corresponding BGP update traffic is a reflection of BGP dynamics taking place in the core of the Internet, where the BGP update volatility is believed to reach maximum rates. Our data spans 8.5 years from mid-2003 through the end of 2011.

### Data pre-processing

- During the period of observation some of the IP addresses of the monitors changed. We identified these changes and concatenated corresponding update time series.

- In the case BGP session between the monitor and the Routeviews collector is broken and re-established, the monitor re-announces all its known paths to the collector producing a burst in the number of BGP updates. These local artifacts of the RouteViews measurement infrastructure are known as *session resets*. Session resets do not represent genuine BGP routing dynamics. We identified and removed BGP updates corresponding to session resets using the method developed in a course of our previous work [2].

- In addition to session resets we have identified and removed BGP updates corresponding to periods of non-stationarity in the dynamics of the monitors, caused by misconfigurations and monitor-specific events. We refer the reader to Ref. [3] for a detailed discussion of these non-stationary periods and the methods used for their identification.

The BGP update datasets used in this work are publicly available at the Figshare repository: `http://figshare.com/articles/Correlation_in_global_routing_dynamics/1549778`.

## III. CORRELATIONS IN BGP UPDATES TIME SERIES

In this section we discuss methods we use to characterize correlations in the BGP update times series: Auto-Correlation Functions (ACF) and Power Spectrum (PS). Linear Detrended Fluctuation Analysis (DFA1) is discussed in the Methods section of the main text.

### A. Auto-Correlation Function

Correlations in the discrete time series $z(t)$ can be quantified by the ACF [4], which is defined as

$$ACF(\tau) = \frac{1}{\sigma^2}\sum_{t=1}^{N-\tau}(z(t)-\mu)(z(t+\tau)-\mu), \tag{1}$$

where $N$ is the total number of elements in the time series, $\mu$ and $\sigma$ are respectively the mean and the standard deviation of the time series:

$$\mu = \frac{1}{N}\sum_{t=1}^{N} z(t), \tag{2}$$

$$\sigma^2 = \frac{1}{N}\sum_{t=1}^{N}\left(z(t)-\mu\right)^2 \tag{3}$$

The values of the ACF range from $+1$ (very high positive correlation) $-1$ (very high negative correlation). In the case there is no correlations, the $ACF(\tau) \approx 0$. In the case $z(t)$ is correlated for up to $t_0$ time steps, corresponding $ACF(\tau)$ is positive for $\tau < t_0$. Among correlated time series one often distinguishes short-range and long-range correlations. In the case of short-range correlations, $ACF$ exhibits a fast, typically exponential, decay to zero:

$$ACF(\tau) \sim e^{-\tau/\zeta}, \tag{4}$$

where $\zeta$ is the effective correlation length. In the case long-range correlations are present, the $ACF$ decreases to zero much slower, typically as a power-law:

$$ACF(\tau) \sim \tau^{-\gamma}. \tag{5}$$

Figure S1**a** depicts the ACF measured for the BGP update time series. All four monitors exhibit power-law scaling of the ACF with $\gamma = 0.5$ of the $NTT$ monitor and $\gamma = 0.4$ for other monitors. Noise in the ACF plots is caused by irregular fluctuations in the time series as well as possible non-stationarities.

### B. Power-Spectrum

Power spectrum is defined as the Fourier Transform of the ACF [4]:

$$S(f) = \int_{-\infty}^{\infty} ACF(\tau)e^{-2\pi i f\tau}\, d\tau \tag{6}$$

In the case of long-range correlations characterized by $ACF \sim \tau^{-\gamma}$ , $S(f)$ also decays as a power-law:

$$S(f) \sim f^{-\beta}, \tag{7}$$

where

$$\beta = 1 - \alpha \tag{8}$$

Even though the Power-Spectrum is fully derived from the ACF, the use of the former in series analysis often helps to decrease the noise. As seen from Fig. S1**b**, $S(f) \sim f^{-\beta}$ for all four monitors with $\beta \approx 0.6$, which is consistent with Eq. (8).

Figures S1**a** and S1**b** complement the results of DFA1, which we report in the main text.

We note the long-range correlation exponents measured with the three methods (ACF ($\gamma$), DFAS1 ($\alpha$) and PSD ($\beta$) conform with expected relations [4–6]:

$$\gamma = 2 - 2\alpha, \tag{9}$$

$$\alpha = \frac{\beta + 1}{2}, \tag{10}$$

$$\gamma = 1 - \beta \tag{11}$$

## IV. RETURN INTERVALS AND MEMORY IN BGP UPDATES

Figure S2**a, c, e, g** displays the distribution of return intervals, $P_q(\tau)$ calculated for all four monitors. Return intervals calculated for different values of the threshold $q$. Upon rescaling $P_q(\tau)$ follow the same master-curve $f(x)$:

$$P_q(\tau) = \frac{1}{\bar{\tau}} f\left(\frac{\tau}{\bar{\tau}}\right), \tag{12}$$

where $f(x)$ does not depend on the threshold value and fits a stretched exponential $exp(-x^{\gamma})$, where $\gamma \approx 0.5$ (Fig. S2**b,d,f,g**). See Supporting Section V for details on data fitting.

We also note that all four monitors exhibit memory effects. As seen, from Fig. S3, large (small) return intervals are likely to be followed by large (small) return intervals.

## V. STATISTICAL TESTS OF DATA FITTING

In this section we present the results on data fitting for the distribution of number of updates, $P(z)$, and the distribution of return intervals, $P_q(\tau)$, calculated for the $NTT$ monitor. For $P(z)$, we fit time series with aggregation window size of 1 min, and for $P_q(\tau)$ we fit return interval time series with $q \in (1, 2, 4, 8, 16, 32)$. Note that we pick these values since they yield a reasonable sample size for performing the fitting.

We tested the goodness of fit using the Kolmogorov-Smirnov (KS) goodness of fit test [7].

The KS test can be summarized as follows. Given two cumulative distribution functions (CDF), $F_1(x)$ and $F_2(x)$ one defines the KS statistic as

$$KS(F_1, F_2) = \sup_x |F_1(x) - F_2(x)|. \tag{13}$$

The KS statistic can be viewed as a separation between the two distribution. The smaller the KS the more similar the two distributions are. One starts with the calculation of the KS statistic for the empirical Cumulative distribution function (CDF) $E(x)$ and the CDF of its best-fit, $BF(x)$, which is referred to as $KS_{eb}$:

$$KS_{eb} \equiv KS(E(x), BF(x)) \tag{14}$$

Then, a large number of data samples $N_s$ is generated using $BF(x)$, each data sample containing a large number of values. For each data sample $i$ one calculates CDF $E_i(x)$ and corresponding KS statistic

$$KS_i \equiv KS(E_i(x), BF(x)) \tag{15}$$

The KS test compares the empirical KS statistic $KS_{eb}$ with the set of synthetic values $KS_i$. Goodness of fit is quantified by the $p$-value by integrating the distribution of synthetic KS values $P(KS)$:

$$p = \int_{KS_{EB}}^{\infty} P(KS) \mathrm{d}KS \tag{16}$$

$p$-values, therefore can be interpreted as the probability that the observed data was the result of its best fit. Large $p$-value indicates that the empirical distribution matches its best fit as good as synthetic data generated from the fit itself, whereas a small $p$-value (typically $p < 0.01$) suggests that the empirical distribution can not be the result of its best fit.

**The distribution of the number of BGP updates.** We employ the maximum-likelihood method proposed by Clauset *et al* [8] to estimate $\mu$ and $z_{min}$ of the $P(z)$ given by

$$P(z) \sim z^{-\mu}, \tag{17}$$

The maximum likelihood estimate yields $\alpha = 2.5$ and $z_{min} \approx 160$. To assess the goodness of fit we get $KS_{eb} \approx 0.014$ and generate $N_s = 10^3$ synthetic data samples each consisting of $10^3$ values (Fig. S4**a**), the resulting $p$-value is $p = 0.992$.

**The distribution of the return intervals obtained for the $NTT$ monitor.**

We argue that the scaled distribution of the number of BGP updates return intervals decays as a stretched exponential distribution in the form $P_q(\tau) \approx e^{-x^{\gamma}}$. In the following we elaborate on confirming that our empirical data is statistically consistent with the best-fit. To find the best fitting distribution, we experimented with fitting the data using the maximum likelihood estimation to various distributions, exponential, power-law, stretched exponential. The latter gives the best-fit with exponent $\gamma \approx 0.5$.

To confirm that the empirical data is statistically consistent with the best-fit, we perform the KS goodness of fit test. We generated $N_s = 10^3$ samples of $10^3$ values each from the stretch exponential fit. We then compute the $P(KS)$ synthetic samples and compared it with $KS_{eb} \approx 0.0395$ (Fig. S4**b**). The obtained $p$-value is $p = 0.08$.

---


[1] Routeviews Project Page;. http://www.routeviews.org.

[2] Zhang B, Kambhampati V, Lad M, Massey D, Zhang L. Identifying BGP routing table transfers. In: MineNet '05: Proceedings of the 2005 ACM SIGCOMM workshop on Mining network data. New York, NY, USA: ACM; 2005. p. 213–218.

[3] Elmokashfi A, Dhamdhere A. Revisiting BGP churn growth. ACM SIGCOMM Computer Communication Review. 2013;44(1):5–12.

[4] Beran J. Statistics for long-memory processes. vol. 61. Chapman and Hall, New York; 1994.

[5] Koscielny-Bunde E, Bunde A, Havlin S, Roman HE, Goldreich Y, Schellnhuber HJ. Indication of a Universal Persistence Law Governing Atmospheric Variability. Phys Rev Lett. 1998 Jul;81:729–732. Available from: http://link.aps.org/doi/10.1103/PhysRevLett.81.729.

[6] Barabasi AL, Stanley HE. Fractal Concepts in Surface Growth. Cambridge University Press; 1995.

[7] Law AM, Kelton DM. Simulation Modeling and Analysis. McGraw-Hill Higher Education; 1999.

[8] Clauset A, Shalizi CR, Newman MEJ. Power-Law Distributions in Empirical Data. SIAM Rev. 2009 Nov;51(4):661–703. Available from: http://dx.doi.org/10.1137/070710111.

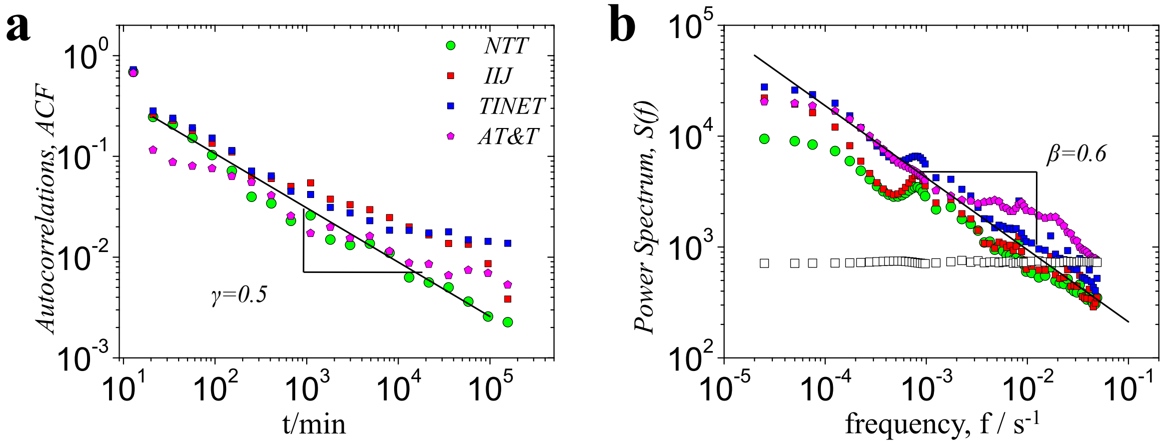


FIG. S1. Correlations in the BGP update times series. **a**, The autocorrelation function, ACF and **b**, The Power Spectrum S(f)

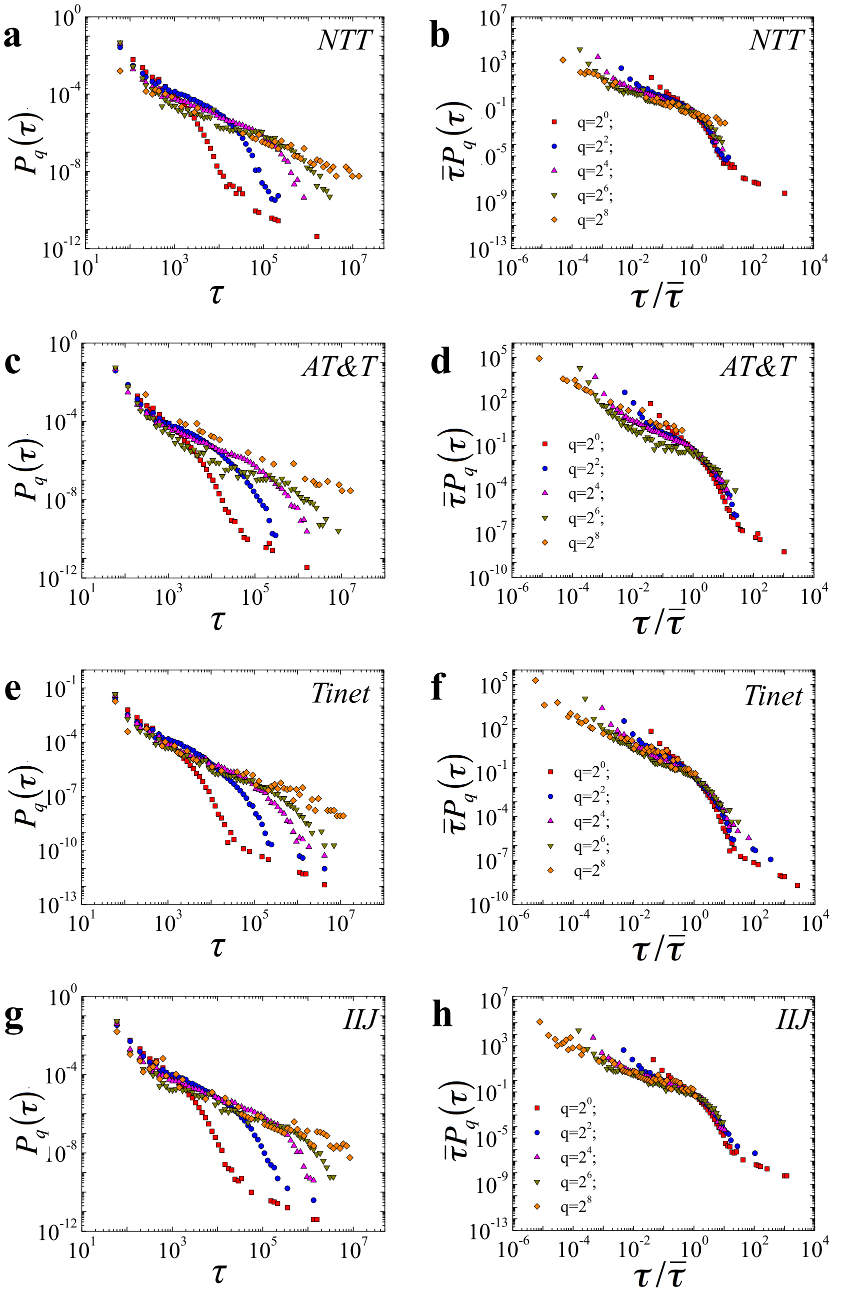


FIG. S2. (Left column) The distribution of return intervals, $P_q(\tau)$, for BGP updates of **a** *NTT*, **c** *AT&T*, **e** *Tinet*, and **g** *IIJ* monitors. the distributions are calculated for different values of threshold $q$. (Right column) Rescaled plots of the BGP return intervals for of **b** *NTT*, **d** *AT&T*, **f** *Tinet*, and **h** *IIJ* monitors.

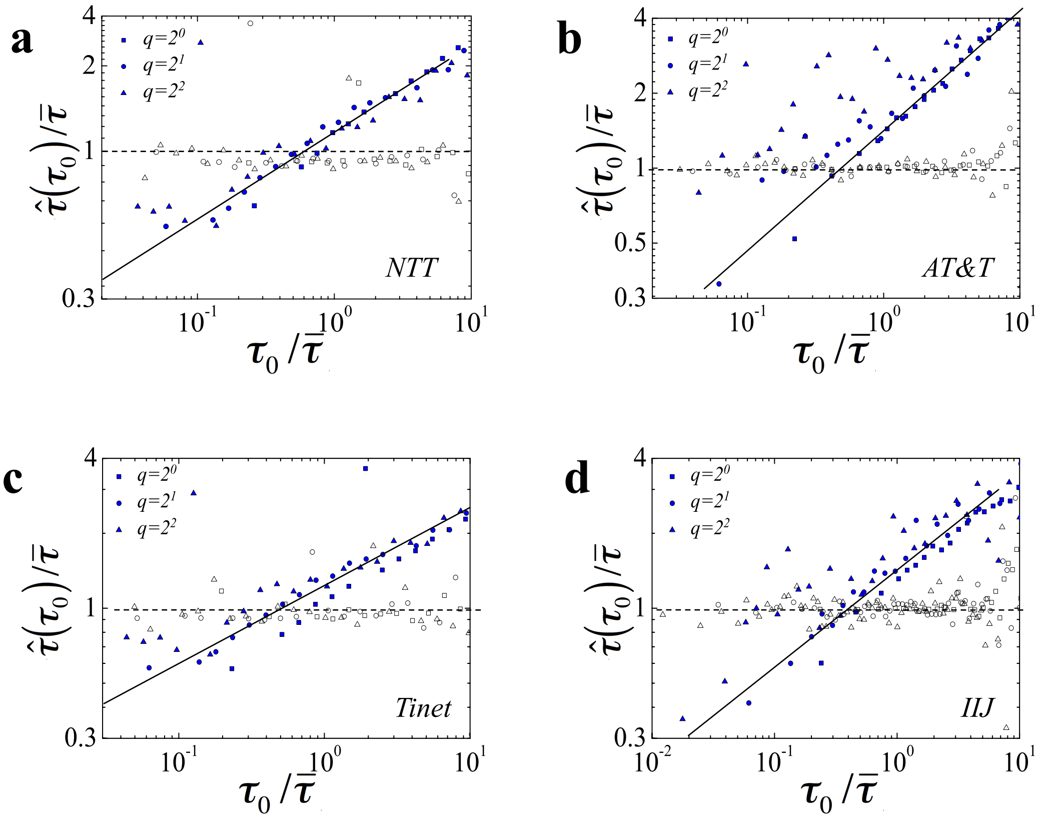


FIG. S3. The mean conditional interval $\hat{\tau}(\tau_0)$ divided by $\overline{\tau}$ as a function of $\frac{\tau_0}{\overline{\tau}}$ for **a** $NTT$, **b** $AT\&T$, **c** $Tinet$, and **d** $IIJ$ monitors. In time series without memory, $\hat{\tau}(\tau_0) = 1$, supported by the open symbols that show the shuffled return interval data.

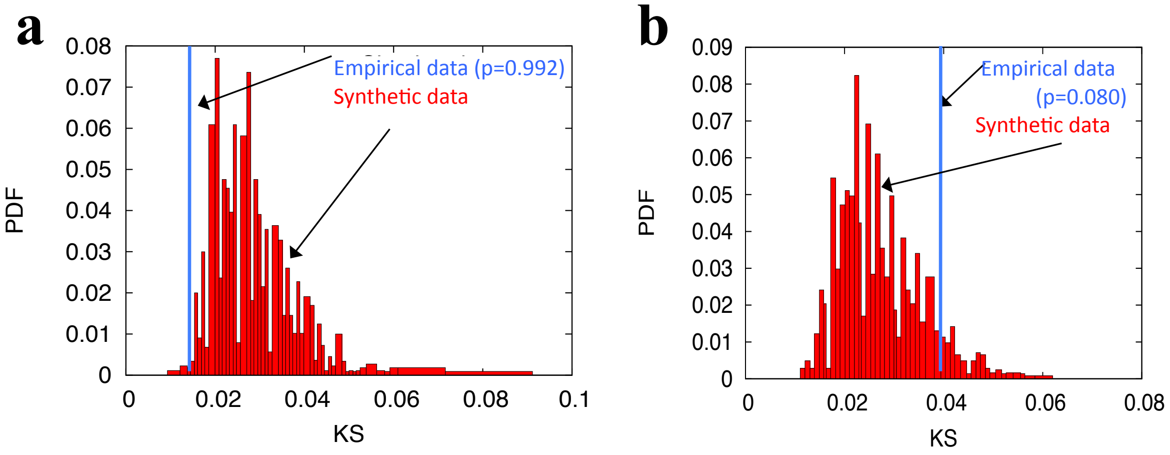


FIG. S4. KS goodness of fit tests for **a**, the distribution of number of BGP updates, $P(z)$ for the $NTT$ monitor, and **b**, the distribution of return intervals $P_q(\tau)$ for the $NTT$ monitor.
