# Supplementary figures and images for: Long-Range Correlations and Memory in the Dynamics of Internet Interdomain Routing
Source: PLoS One. 2015 Nov 3;10(11):e0141481. doi: 10.1371/journal.pone.0141481 (PMC4631327; doi:10.1371/journal.pone.0141481)

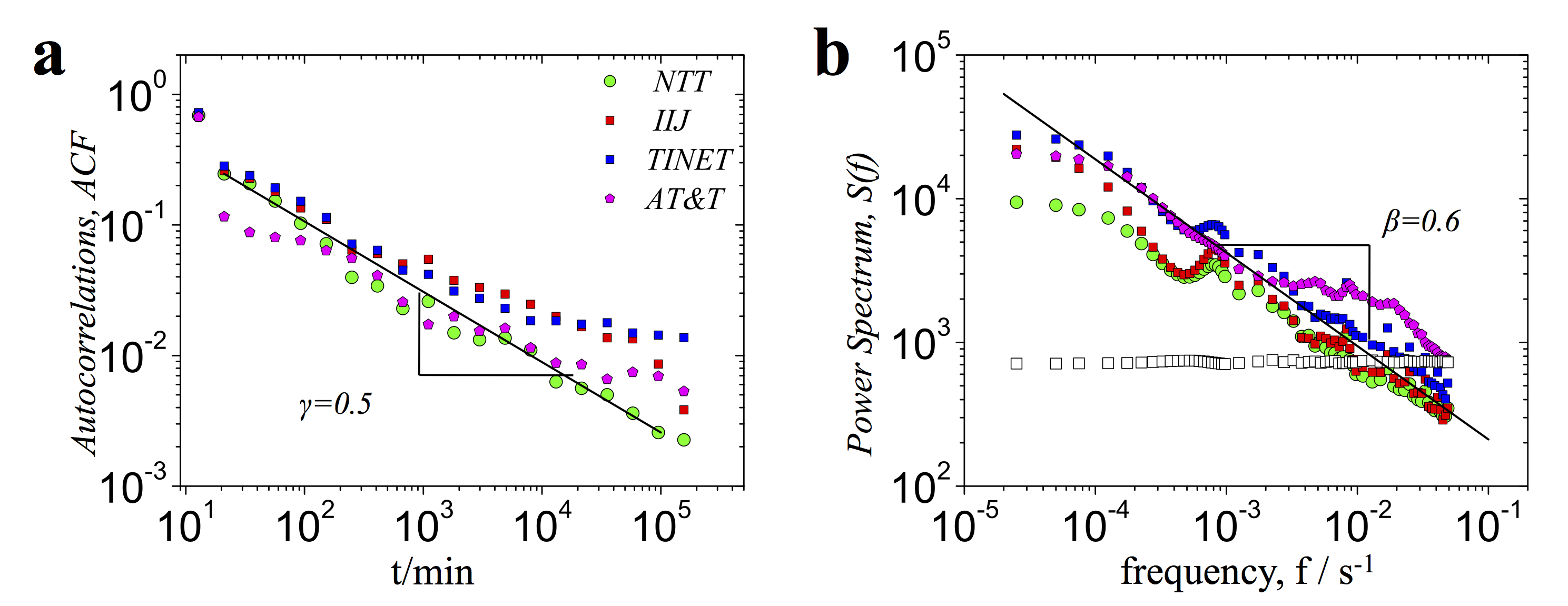

Supplement: S1 Fig — a, The autocorrelation function, ACF and b, The Power Spectrum S(f). (TIFF) [file pone.0141481.s002.tiff]

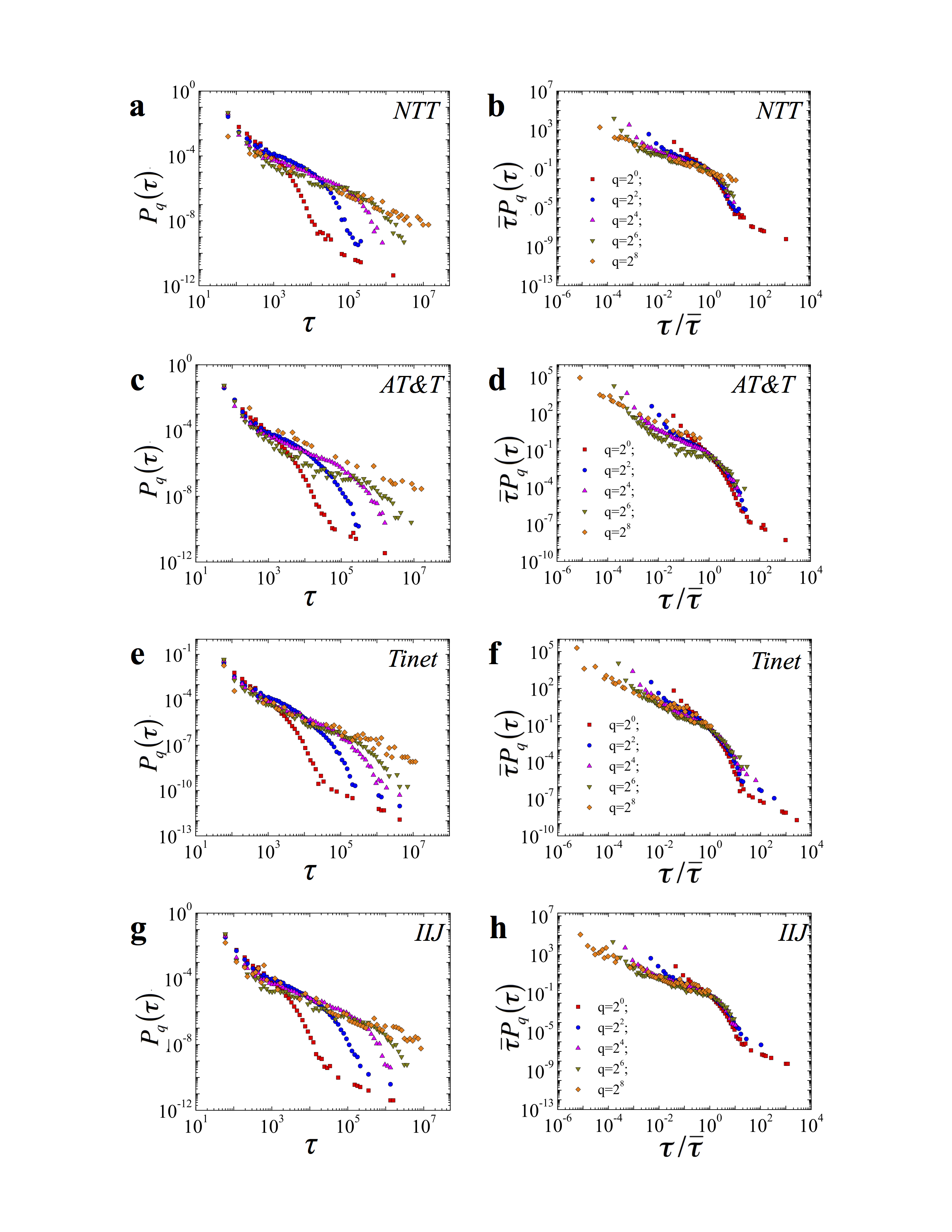

Supplement: S2 Fig — (Left column) The distribution of return intervals, P q(τ), for BGP updates of a, NTT, c, AT&T, e, Tinet, and g, IIJ monitors. The distributions are calculated for different values of threshold q. (Right column) Rescaled plots of the BGP return intervals of b, NTT, d, AT&T, f, Tinet, and h, IIJ monitors. (TIFF) [file pone.0141481.s003.tiff]

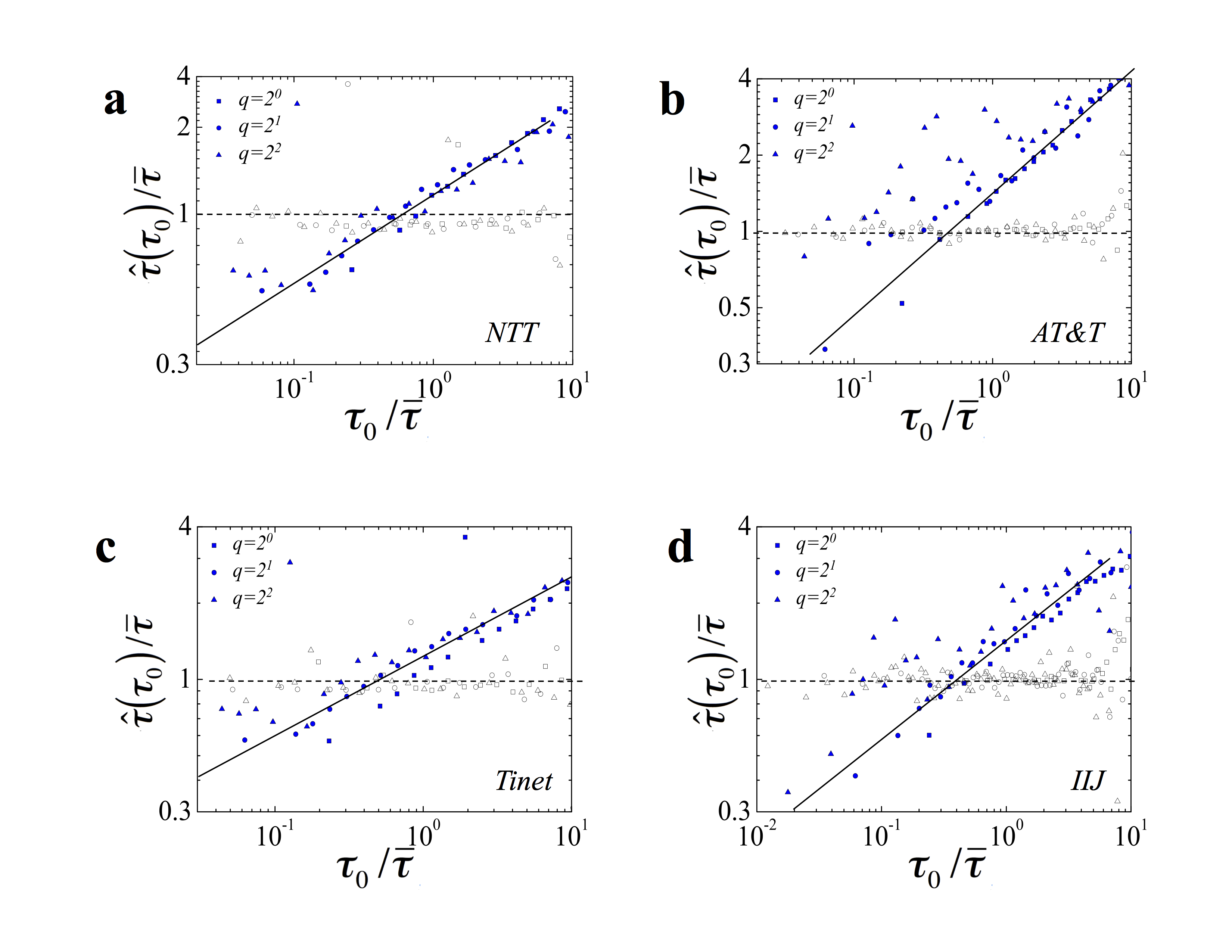

Supplement: S3 Fig — The mean conditional interval τ^(τ0) divided by τ¯ as a function of τ0τ¯ for a NTT, b AT&T, c Tinet, and d IIJ monitors. In time series without memory, τ^(τ0)=1, indicated by the open symbols that show the shuffled return interval data. (TIFF) [file pone.0141481.s004.tiff]

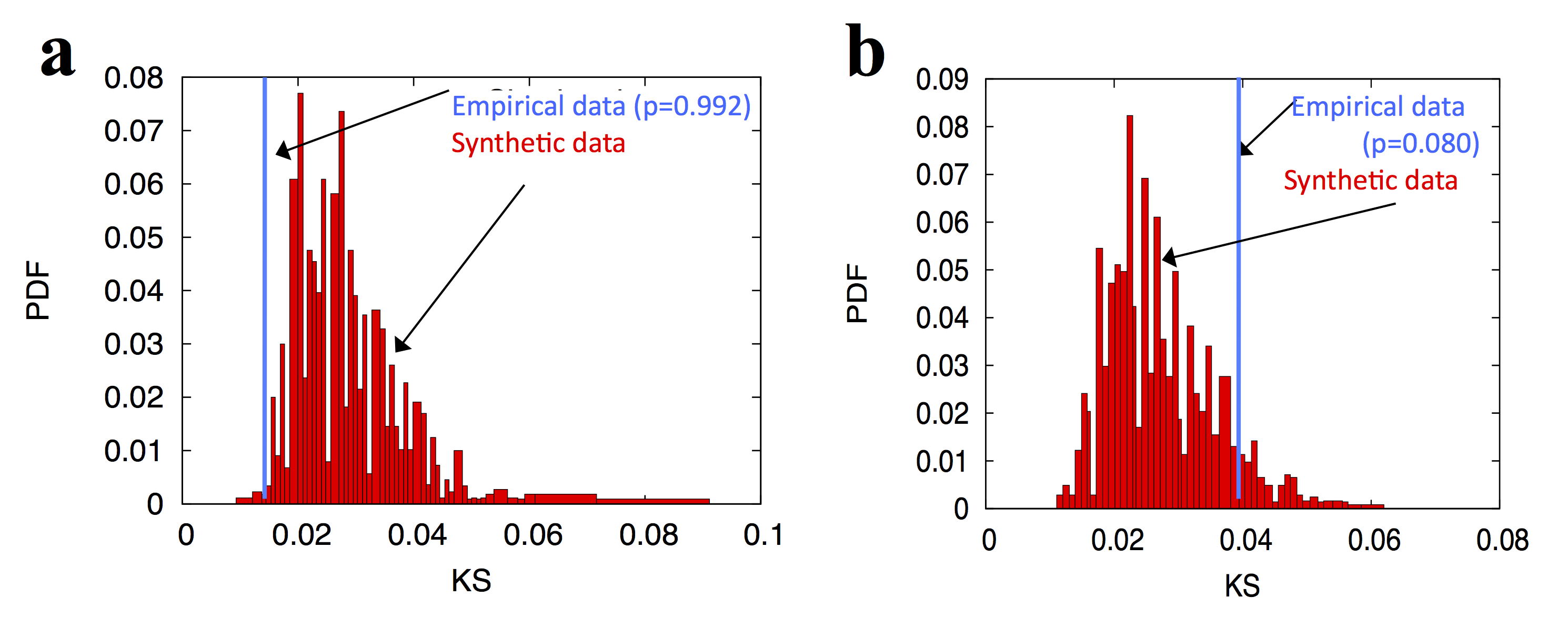

Supplement: S4 Fig — KS goodness of fit tests for a, the distribution of number of BGP updates, P(z) for the NTT monitor, and b, the distribution of return intervals P q(τ) for the NTT monitor. (TIFF) [file pone.0141481.s005.tiff]
